# Supplementary material for: Single-cell analysis of Barrett’s esophagus and carcinoma reveals cell types conferring risk via genetic predisposition
Source: Cell Genom. 2025 Sep 8;5(10):100980. doi: 10.1016/j.xgen.2025.100980 (PMC12790991; doi:10.1016/j.xgen.2025.100980)
Supplement: Document S1. Figures S1–S12 and Tables S1, S3, S5, and S8–S10 [file mmc1.pdf]

Cell Genomics, Volume *5*

Supplemental information

Single-cell analysis of Barrett's esophagus and carcinoma reveals cell types conferring risk via genetic predisposition

Marten C. Wenzel, Pouria Dasmeh, Patrick S. Plum, Ann-Sophie Giel, Sascha Hoppe, Marek Franitza, Christoph Jonas, René Thieme, Yue Zhao, Dominik Heider, Claire Palles, Rebecca Claire Fitzgerald, Christiane J. Bruns, Reinhard Buettner, Alexander Quaas, Ines Gockel, Carlo Maj, Seung-Hun Chon, Johannes Schumacher, and Axel M. Hillmer

Single cell analysis of Barrett's esophagus and carcinoma reveals cell types conferring risk via genetic predisposition

Marten C. Wenzel, Pouria Dasmeh, Patrick S. Plum, Ann-Sophie Giel,
Sascha Hoppe, Marek Franitza, Christoph Jonas, René Thieme,
Yue Zhao, Dominik Heider, Claire Palles, Rebecca Claire Fitzgerald,
Christiane J. Bruns, Reinhard Buettner, Alexander Quaas, Ines Gockel, Carlo Maj,
Seung-Hun Chon, Johannes Schumacher, Axel M. Hillmer

Content

Supplementary Tables 2
Supplementary Figures 11
Supplementary References 23

Supplementary Tables

Supplementary Table 1. Patient information is shown for BE and EAC patients, related to Figure 1.

Patient ID	Diagnosis	Gender	Age	Segment length (BE) [cm]	Dysplasia present (BE)	Tumor localization (EAC)	Tissues analyzed
BE_pat01	BE	m	67	2	no	-	EN, GFN, BE
BE_pat02	BE	m	61	3	no	-	EN, GFN, BE
BE_pat03	BE	m	46	2	no	-	GFN, BE *
BE_pat04	BE	m	70	8	yes	-	EN, GFN, BE
BE_pat05	BE	m	63	3	no	-	EN, GFN, BE
EAC_pat01	EAC	m	70	-		AEG I	EN, GFN, EAC
EAC_pat02	EAC	m	76	-		AEG I	EN, GFN, EAC
EAC_pat03	EAC	m	87	-		AEG II	EN, GFN, EAC
EAC_pat04	EAC	m	70	-		AEG II	EN, GFN, EAC

* EN tissue of BE_pat03 did not result in successful preparation of NGS library

AEG - adenocarcinomas of the esophagogastric junction

AEG I - adenocarcinoma of the distal esophagus

AEG II - true adenocarcinoma of the cardia

AEG III - subcardial adenocarcinoma

Supplementary Table 3. Full gene names are shown for discussed genes, related to Figure 2.

ACKR1	Atypical chemokine receptor 1
ACKR2	Atypical chemokine receptor 2
ACTA2	alpha smooth muscle actin
CCl11	chemokine (C-C motif) ligand 11
CCL2	chemokine (C-C motif) ligand 2
CCL4	chemokine (C-C motif) ligand 4
CCL5	chemokine (C-C motif) ligand 5
CD79A	B-cell antigen receptor complex-associated protein alpha chain
CEACAM5	Carcinoembryonic antigen-related cell adhesion molecule 5
CEACAM6	Carcinoembryonic antigen-related cell adhesion molecule 6
CHGA	Chromogranin A
DCN	Decorin
DPT	Dermatopontin
EPCAM	Epithelial cell adhesion molecule
FPR1	Formyl peptide receptor 1
FRZB	Secreted frizzled-related protein 3
HNF4A	Hepatocyte nuclear factor 4 alpha
ITLN1	Intelectin-1
KRT20	Keratin 20
KRT6B	Keratin 6B
KRT7	Keratin 7
LEFTY1	Left-right determination factor 1
LIPF	Gastric lipase
LUM	Lumican
MUC2	Mucin 2
MUC5AC	Mucin-5AC
MYL9	Myosin regulatory light polypeptide 9
NEUROG3	Neurogenin-3
NRXN1	Neurexin-1-alpha
OLFM4	Olfactomedin 4
PDGFRA	Platelet-derived growth factor receptor A
PECAM1	Platelet endothelial cell adhesion molecule
PGA3	Pepsinogen 3
PGA4	Pepsinogen 4
PGA5	Pepsinogen 5
PGC	Progastricsin
PHGR1	Proline, Histidine And Glycine Rich 1
PLP1	Proteolipid protein 1
PLVAP	Plasmalemma vesicle-associated protein
REG4	Regenerating islet-derived protein 4
RGS5	Regulator of G-protein signaling 5
S100A8	S100 calcium-binding protein A8
S100A9	S100 calcium-binding protein A9
SFRP1	Secreted frizzled-related protein 1
SFRP2	Secreted frizzled-related protein 2
SPINK4	Serine Peptidase Inhibitor Kazal Type 4
TAGLN	Transgelin
TFF1	Trefoil factor 1
TFF2	Trefoil factor 2
TFF3	Trefoil factor 3
VWF	Von Willebrand factor
WNT11	Protein Wnt-11

Supplementary Table 5. Somatic copy number alterations (SCNAs) derived from scRNA-seq of tumor samples are compared to recurrent SCNAs observed in EAC, related to Figure 3.

Patient/study	Copy gains	Copy losses
EAC-01	1q, 2p, 2q, 6p, 7p, 8q, 11q, 12p, 19q, 20q	3p, 4q, 5q, 6p, 13q, 17p
EAC-02	6p, 8q, 9q	6q, 9q
EAC-03	6p, 14q	1q, 6q, 9q
EAC-04	1p, 3q, 6p, 6q, 8q, 9q, 11q, 12p, 12q, 17q, 20p, 20q	1p, 1q, 3p, 4p, 4q, 8p, 15q, 17p, 18q, 19q, 22q
Pasello et al. [1]	6q, 7p, 7q, 8q, 11q, 15q, 17q	1p, 3p, 4p, 4q, 5q, 8p, 9p, 17p, 18q
Frankell et al. [2*]	1p36.22, 1q21.1, 1q21.1, 1q21.3, 1q22, 3q26.2, 3q29, 6p21.1, 6p21.32, 6p21.32, 6p21.33, 6p22.1, 7q21.3, 7q22.1, 8p23.1, 8q24.13, 8q24.3, 9q34.3, 11q13.3, 11q14.1, 12p11.23, 12q15, 13q14.11, 14q11.2, 17q11.2, 17q12, 17q21.2, 18q11.2,	1p21.2, 1p36.11, 1p36.13, 1p36.21, 21p11.1, 21q11.2, 22q11.21, 22q11.21, 22q13.32, 3q11.2, 4q35.1, 5q12.1, 5q23.1, 6p21.32, 6p21.32, 6p24.2, 7q36.2, 8p23.3, 9p13.1, 9p21.3, 9p24.1, 9q13, 10q23.31, 10q26.2, 11q25, 14q11.2, 14q32.33, 14q32.33, 15q11.1, 15q11.2, 15q13.1, 16p11.2, 16p13.3, 16q12.1, 16q23.1, 17p11.2,

* GISTIC peaks with q value < 10^{-10}

Supplementary Table 8. Partitioned heritability analysis is performed using LDSR analysis to assess the enrichment of lung cancer risk variants, related to Figure 5.

GWAS summary statistics for lung cancer in never-smokers (2,355 cases and 7,504 controls of European ancestry; GWAS Catalog study GCST004747) were used.

Name	Enrichment p-value	Coefficient	Coefficient_z.score
Chief.Cells	0.11	-2.20E-08	-1.583131346
Generic.myofibroblasts.CAFs	0.16	-2.21E-08	-1.400464594
Esophageal.fibroblasts.2	0.19	-1.91E-08	-1.318026961
Gastric.endothelial.cells	0.23	-1.63E-08	-1.215147147
CAFs	0.30	1.51E-08	1.028428569
Intestinal.metaplasia.cells	0.32	-1.56E-08	-1.00008376
Basophils	0.34	1.60E-08	0.963848552
CD8..NKT.like.cells	0.38	-1.38E-08	-0.871293014
Endothelial	0.40	1.28E-08	0.840433647
Esophagus.epithelial.cells	0.41	-1.29E-08	-0.822396182
EAC.04	0.43	1.43E-08	0.79764506
Misclassified	0.46	1.15E-08	0.744803124
Esophageal.endothelial.cells.1	0.50	-9.85E-09	-0.674849749
Esophageal.endothelial.cells.2	0.52	-8.93E-09	-0.648565873
Foveolar.cells.2	0.54	-9.26E-09	-0.614182003
Plasma.B.cells	0.56	-9.12E-09	-0.578948392
Lymphatic.endothelial.cells	0.60	-7.67E-09	-0.526319163
Parietal.cells	0.61	-7.67E-09	-0.509971215
Neuronal.cells	0.61	6.53E-09	0.506276423
Macrophages	0.62	-8.22E-09	-0.500098441
EAC.02	0.64	-7.19E-09	-0.473661221
Classical.Monocytes	0.75	-4.89E-09	-0.312983782
Esophageal.fibroblasts.1	0.79	3.53E-09	0.265935665
Enteroendocrine.Cells	0.79	3.97E-09	0.26045547
Pre.B.cells	0.84	-3.15E-09	-0.197721547
Gastric.fibroblasts.2	0.85	2.60E-09	0.188498256
BE.fibroblasts	0.86	-2.65E-09	-0.18260363
Plasmacytoid.dendritic.cells	0.86	3.03E-09	0.179952266
Foveolar.cells.1	0.86	-2.82E-09	-0.173935775
EAC.01	0.87	-2.82E-09	-0.168530121
Gastric.fibroblasts.1	0.89	-2.07E-09	-0.137817098
EAC.03	0.90	-2.02E-09	-0.122298855
Memory.CD4..T.cells	0.93	-1.36E-09	-0.090460055

Supplementary Table 9. Gene-prioritization of GWAS associated loci based on single-cell specific expression is shown for associated cell types for EAC, related to Figure 5.

Genomic Locus	Leading variant	Gwas *p*	Genes in the LD block	Prioritized genes (Cell types, Expression specificity)	Non-prioritized genes
4	rs376563	2.013e-09	FNDC1, SOD2, ACAT2, TCP1, MRPL18, PNLDC1, MAS1, IGF2R, SLC22A1, SLC22A2, SLC22A3, LPA, PLG, AGPAT4, PARK2, PACRG	PLG (GFN_Fibroblasts_1, 1), PNLDC1 (Macrophages, 0.766), MAS1 (Endothelial, 0.711), PACRG (GFN_Fibroblasts_1, 0.652), SLC22A1 (Memory.CD4..T.Cells, 0.62), FNDC1 (Endothelial, 0.577), SLC22A3 (GFN_Fibroblasts_2, 0.455), IGF2R (Plasmacytoid.Dendritic.Cells, 0.283), MRPL18 (Endothelial, 0.237), TCP1 (Endothelial, 0.183), AGPAT4 (GFN_EC, 0.152), SOD2 (GFN_Fibroblasts_1, 0.046)	ACAT2, SLC22A2, LPA, PARK2
7	rs3111601	8.087e-09	C16orf74, EMC8, COX4I1, IRF8, FOXF1, MTHFSD	IRF8 (Plasmacytoid.Dendritic.Cells, 0.659), FOXF1 (GFN_Fibroblasts_2, 0.31), MTHFSD (GFN_Fibroblasts_2, 0.28), C16ORF74 (Endothelial, 0.187), EMC8 (ESO_EC_2, 0.148), COX4I1 (Classical.Monocytes, 0.012)	
5	rs2188554	1.228e-08	TFEC, CAV2, MET, ST7, ST7-OT4, AC106873.4, WNT2, ASZ1, CFTR, CTTNBP2, NAA38, ANKRD7	ANKRD7 (CAFs, 0.691), CFTR (ESO_EC_2, 0.597), WNT2 (EN_Fibroblasts_1, 0.423), TFEC (Endothelial, 0.365), ST7-OT4 (GFN_Fibroblasts_2, 0.342), MET (ESO_EC_2, 0.285), CAV2 (GFN_EC, 0.267), ST7 (Endothelial, 0.181), CTTNBP2 (Basophils, 0.024)	AC106873.4, ASZ1, NAA38
3	rs13115814	1.304e-08	IL21, BBS12, FGF2, NUDT6, SPATA5, SPRY1, ANKRD50, FAT4	IL21 (ESO_EC_2, 0.986), SPRY1 (GFN_EC, 0.497), FAT4 (GFN_Fibroblasts_2, 0.373), ANKRD50 (GFN_EC, 0.312), NUDT6 (ESO_EC_2, 0.272), FGF2 (GFN_EC, 0.206)	BBS12, SPATA5
6	rs10431648	2.196e-08	SCFD1, COCH, STRN3, AKAP6	AKAP6 (GFN_Fibroblasts_2, 0.244), STRN3 (GFN_Fibroblasts_2, 0.186), COCH (GFN_EC, 0.164)	SCFD1
1	rs10193919	2.68e-08	SMC6, MSGN1, TTC32, PUM2, RHOB,	TDRD15 (Basophils, 0.979), GDF7 (Endothelial, 0.675), APOB (CAFs, 0.56), SMC6 (Plasmacytoid.Dendritic.Cells, 0.193), TTC32 (GFN_EC,	MSGN1, PUM2, HS1BP3, C2ORF43

			HS1BP3, GDF7, C2orf43, APOB, TDRD15, KLHL29	0.13), RHOB (GFN_Fibroblasts_2, 0.124), KLHL29 (EN_Fibroblasts_1, 0.057)	
2	rs9880983	4.266e-08	YEATS2, MAP6D1, PARL, ABCC5, HTR3D, HTR3C, HTR3E, DVL3, AP2M1, ABCF3, VWA5B2, ALG3, ECE2, CAMK2N2, PSMD2	CAMK2N2 (ESO_EC_2, 0.905), VWA5B2 (Basophils, 0.593), ABCC5 (Endothelial, 0.282), ABCF3 (GFN_EC, 0.282), MAP6D1 (GFN_Fibroblasts_2, 0.259), PARL (Endothelial, 0.215), PSMD2 (GFN_Fibroblasts_2, 0.154), ALG3 (GFN_Fibroblasts_1, 0.039)	YEATS2, HTR3D, HTR3C, HTR3E, DVL3, AP2M1, ECE2

Supplementary Table 10. Gene-prioritization of GWAS associated loci based on single-cell specific expression is shown for associated cell types for BE, related to Figure 5.

Genomic Locus	Leading variant	Gwas *p*	Genes in the LD block	Prioritized genes (Cell types, Expression specificity)	Non-prioritized genes
7	rs2504930	5.354e-14	FNDC1, SOD2, WTAP, ACAT2, TCP1, MRPL18, PNLDC1, MAS1, IGF2R, SLC22A1, SLC22A2, SLC22A3, LPA, PLG, AGPAT4, PARK2, PACRG	SLC22A2 (Generic.Myofibroblasts.CAFs, 1), SOD2 (Classical.Monocytes, 0.501), WTAP (Classical.Monocytes, 0.354), ACAT2 (Classical.Monocytes, 0.292), PACRG (GFN_Fibroblasts_1, 0.254), AGPAT4 (Classical.Monocytes, 0.248), SLC22A1 (ESO_EC_1, 0.172), MAS1 (ESO_EC_2, 0.15), PNLDC1 (BE_Fibroblasts, 0.123), FNDC1 (Endothelial, 0.045), SLC22A3 (GFN_Fibroblasts_1, 0.037)	TCP1, MRPL18, IGF2R, LPA, PLG, PARK2
6	rs72760500	3.15e-13	C7, MROH2B, C6, PLCXD3, OXCT1, ANXA2R, ZNF131, NIM1K, HMGCS1, CCL28, C5orf28, C5orf34, PAIP1, NNT, FGF10, MRPS30, HCN1	NIM1K (Neuronal.Cells, 0.835), FGF10 (Misclassified, 0.581), CCL28 (Basophils, 0.532), PLCXD3 (Endothelial, 0.486), ANXA2R (Basophils, 0.486), OXCT1 (Basophils, 0.279), HMGCS1 (Classical.Monocytes, 0.267), ZNF131 (Basophils, 0.21), NNT (Basophils, 0.167), MRPS30 (Classical.Monocytes, 0.148), C5ORF34 (GFN_Fibroblasts_2, 0.05), C7 (ESO_EC_1, 0.01)	MROH2B, C6, C5ORF28, PAIP1, HCN1
1	rs3072	3.514e-11	SMC6, MSGN1, TTC32, PUM2, RHOB, HS1BP3, GDF7, C2orf43, APOB, TDRD15, KLHL29	KLHL29 (Neuronal.Cells, 0.479), PUM2 (Basophils, 0.294), HS1BP3 (Basophils, 0.27), TTC32 (Misclassified, 0.201), RHOB (Neuronal.Cells, 0.188), APOB (BE_Fibroblasts, 0.17), GDF7 (Generic.Myofibroblasts.CAFs, 0.139), SMC6 (Generic.Myofibroblasts.CAFs, 0.008)	MSGN1, C2ORF43, TDRD15
10	rs1441815	3.628e-11	MNS1, GCOM1, MYZAP, POLR2M, ALDH1A2, AQP9, LIPC, ADAM10, FAM63B, RNF111, CCNB2, C15ORF31, GCNT3,	MYZAP (Lymphatic.Endothelial.Cells, 0.909), AQP9 (Classical.Monocytes, 0.852), GCOM1 (Lymphatic.Endothelial.Cells, 0.828), LIPC (Misclassified, 0.585), MNS1 (Neuronal.Cells, 0.363), RNF111 (Basophils, 0.307), ALDH1A2 (Misclassified, 0.292), POLR2M (Basophils, 0.205), ADAM10	FAM63B, C15ORF31, GCNT3, GTF2A2, FOXB1

			GTF2A2, BNIP2, FOXB1	(Classical.Monocytes, 0.149), BNIP2 (ESO_EC_2, 0.055), CCNB2 (CD8..NKT.like.cells, 0.015)	
8	rs560993129	6.131e-11	ERI1, MSRA, PRSS55, RP1L1, C8orf74, SOX7, PINX1, XKR6, AF131215.5, MTMR9, SLC35G5, C8orf12, FAM167A, BLK, GATA4, C8orf49, NEIL2, FDFT1, RP11-297N6.4, CTSB, DEFB136, DEFB135, DEFB134, RP11-481A20.11, USP17L2, FAM86B1, DEFB130, FAM86B2	RP1L1 (Endothelial, 0.945), XKR6 (Basophils, 0.787), GATA4 (Endothelial, 0.709), FAM86B1 (Misclassified, 0.646), MSRA (Basophils, 0.6), SLC35G5 (GFN_Fibroblasts_2, 0.432), AF131215.5 (Memory.CD4..T.Cells, 0.184), SOX7 (ESO_EC_2, 0.167), CTSB (Classical.Monocytes, 0.154), FDFT1 (Classical.Monocytes, 0.145), NEIL2 (Misclassified, 0.141), ERI1 (Neuronal.Cells, 0.139), PINX1 (Classical.Monocytes, 0.134), BLK (Endothelial, 0.07), MTMR9 (ESO_EC_2, 0.06), FAM167A (Macrophages, 0.039)	PRSS55, C8ORF74, C8ORF12, C8ORF49, RP11-297N6.4, DEFB136, DEFB135, DEFB134, RP11-481A20.11, USP17L2, DEFB130, FAM86B2
9	rs7045553	2.003e-10	ZNF782, HIATL2, CCDC180, TMOD1, TSTD2, NCBP1, FOXE1, CORO2A	TMOD1 (Basophils, 0.568), CCDC180 (Neuronal.Cells, 0.541), CORO2A (Basophils, 0.461), TSTD2 (Basophils, 0.243), ZNF782 (Misclassified, 0.215), NCBP1 (Classical.Monocytes, 0.207)	HIATL2, FOXE1
11	rs35631104	4.192e-10	MCTP2, AC016251.1, AC087477.1, LINC00923	LINC00923 (Lymphatic.Endothelial.Cells, 0.964), MCTP2 (Basophils, 0.475)	AC016251.1, AC087477.1
2	rs146917555	1.759e-09	GTF3C3, C2orf66, MARS2, BOLL, PLCL1, SATB2, FTCDNL1, C2orf69	MARS2 (Basophils, 0.612), C2ORF66 (Memory.CD4..T.Cells, 0.269), GTF3C3 (Basophils, 0.24), FTCDNL1 (Neuronal.Cells, 0.231), C2ORF69 (Classical.Monocytes, 0.18), SATB2 (Neuronal.Cells, 0.17), PLCL1 (Generic.Myofibroblasts.CAFs, 0.026)	BOLL
3	rs559880479	2.828e-09	FOXP1, EIF4E3, GPR27, PROK2, GXYLT2, PPP4R2,	GXYLT2 (Misclassified, 0.397), EIF4E3 (Classical.Monocytes, 0.329), FOXP1 (Basophils, 0.297), PDZRN3 (Misclassified, 0.244), EBLN2 (Classical.Monocytes, 0.217),	PROK2

			EBLN2, PDZRN3	PPP4R2 (Neuronal.Cells, 0.116), GPR27 (Macrophages, 0.099)	
4	rs13124203	3.089e-09	IL21, BBS12, NUDT6, SPATA5, SPRY1, ANKRD50, FAT4	BBS12 (Neuronal.Cells, 0.546), SPATA5 (Basophils, 0.531), ANKRD50 (Neuronal.Cells, 0.234), NUDT6 (Lymphatic.Endothelial.Cells, 0.101), SPRY1 (ESO_EC_2, 0.067), FAT4 (GFN_Fibroblasts_2, 0.067), IL21 (Memory.CD4..T.Cells, 0.011)	
12	rs3950627	4.654e-09	CTC-786C10.1, C16orf74, EMC8, COX4I1, IRF8, FOXF1, MTHFSD, FOXC2, C16orf95	FOXC2 (Lymphatic.Endothelial.Cells, 0.391), C16ORF74 (Endothelial, 0.301), COX4I1 (Basophils, 0.189), IRF8 (Classical.Monocytes, 0.179), FOXF1 (Misclassified, 0.174), C16ORF95 (Endothelial, 0.073), EMC8 (Lymphatic.Endothelial.Cells, 0.062), MTHFSD (ESO_EC_2, 0.055)	CTC-786C10.1
5	rs42202	9.079e-09	IRX2, C5orf38, KIAA0947, ADCY2	ADCY2 (GFN_Fibroblasts_2, 0.348), C5ORF38 (GFN_EC, 0.189), IRX2 (Lymphatic.Endothelial.Cells, 0.095)	KIAA0947

Supplementary Figures

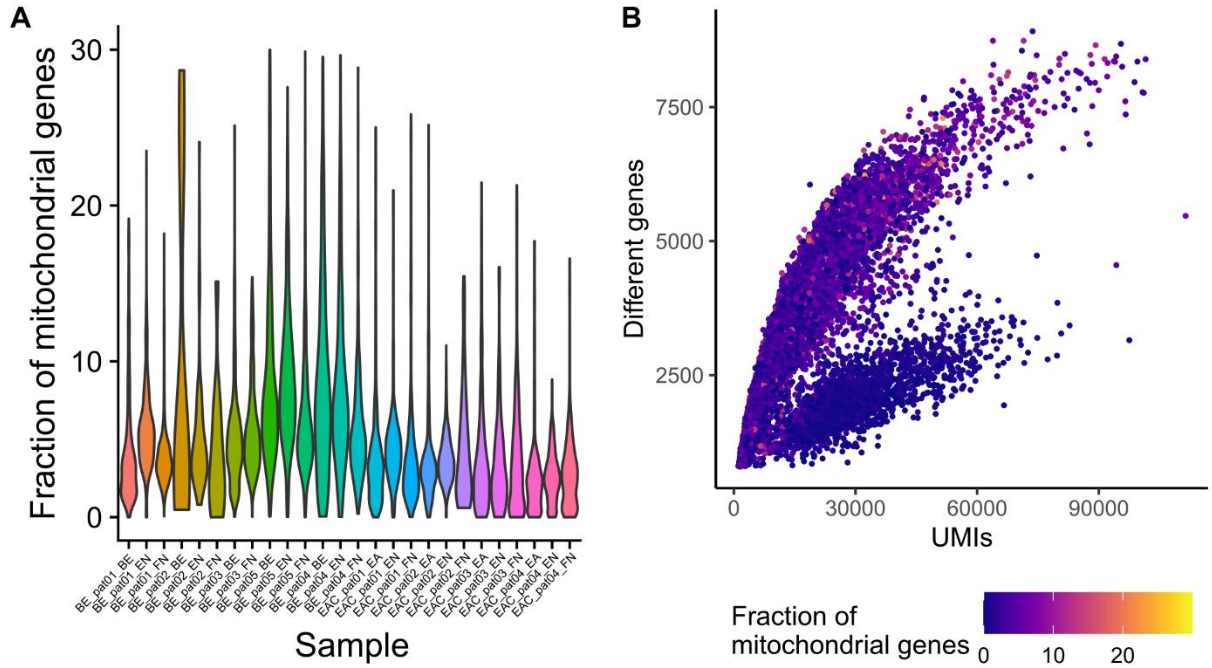

Supplementary Figure 1. Quality Control plots showing fraction of mitochondrial genes and number of genes, related to Figure 1. A Percentage of mitochondrial genes regarding the total number of molecules per sample. **B** Number of different genes as a function of the total number of UMIs. Notably, the population of cells following the lower saturation curve consists of plasma B cells from different patients only.

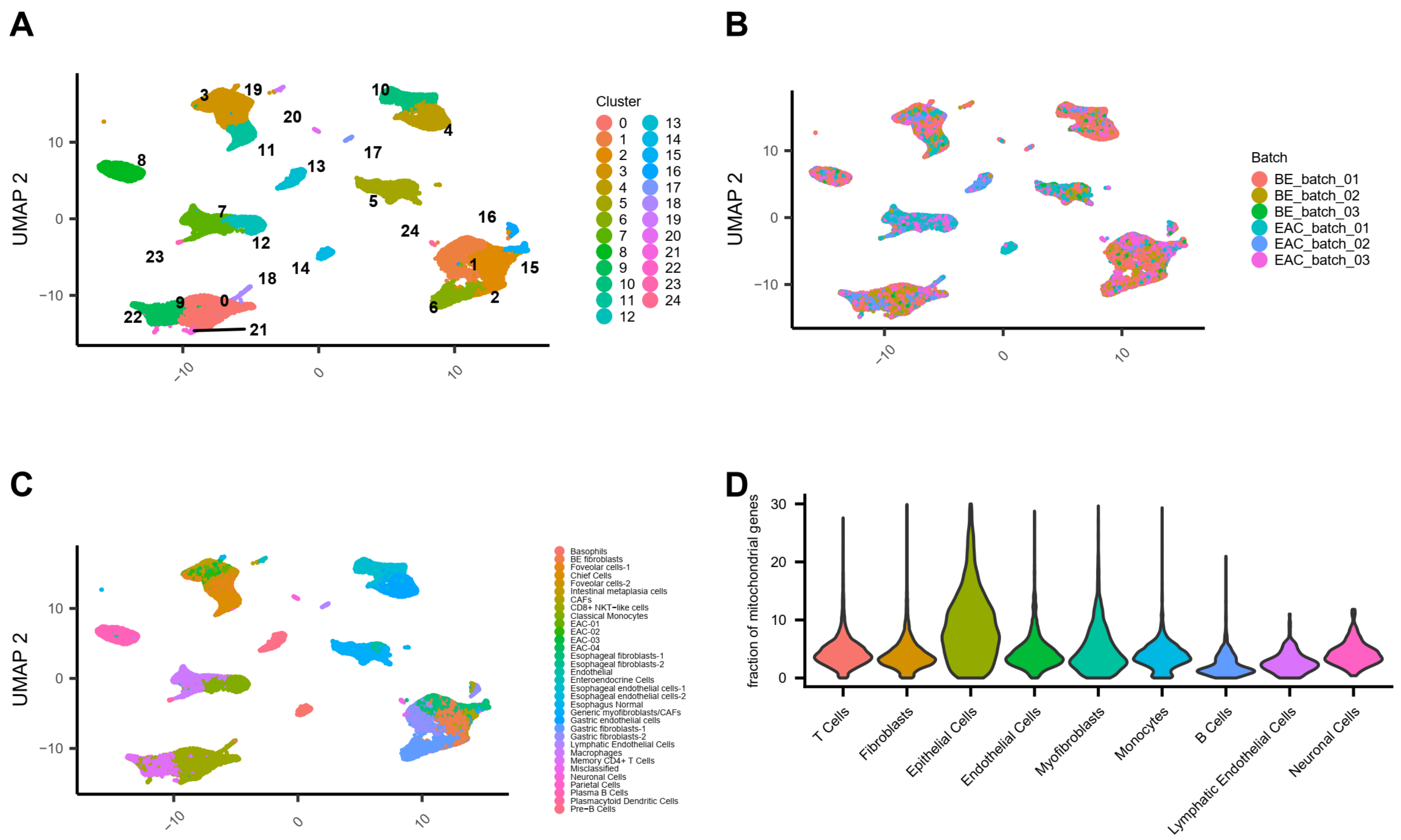

Supplementary Figure 2. Single cell clusters are characterized, related to Figure 1. A UMAP representation of the whole data set showing initial clusters. **B** UMAP representation with color coding for batch origin. **C** UMAP representation with color coding for subclusters. **D** Percentage of mitochondrial genes as a function of cell type family.

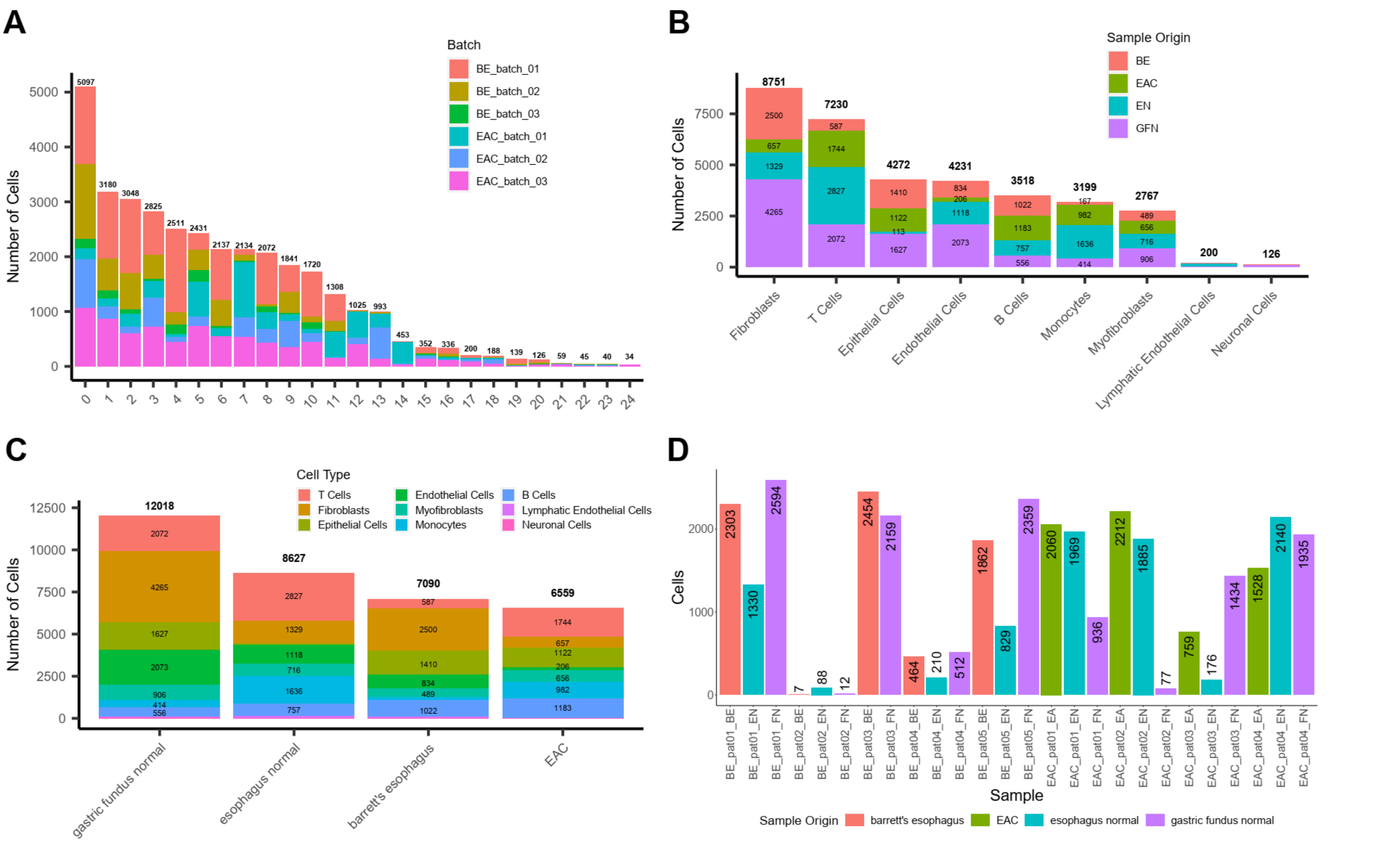

Supplementary Figure 3. Single cell clusters and tissue of origin are characterized, related to Figure 1. A Composition of the initial clusters regarding batch origin to show batch effect control. **B** Contribution of sample origin to the cell type family. **C** Number of cells from different cell type families per sample type. **D** Cells per sample.

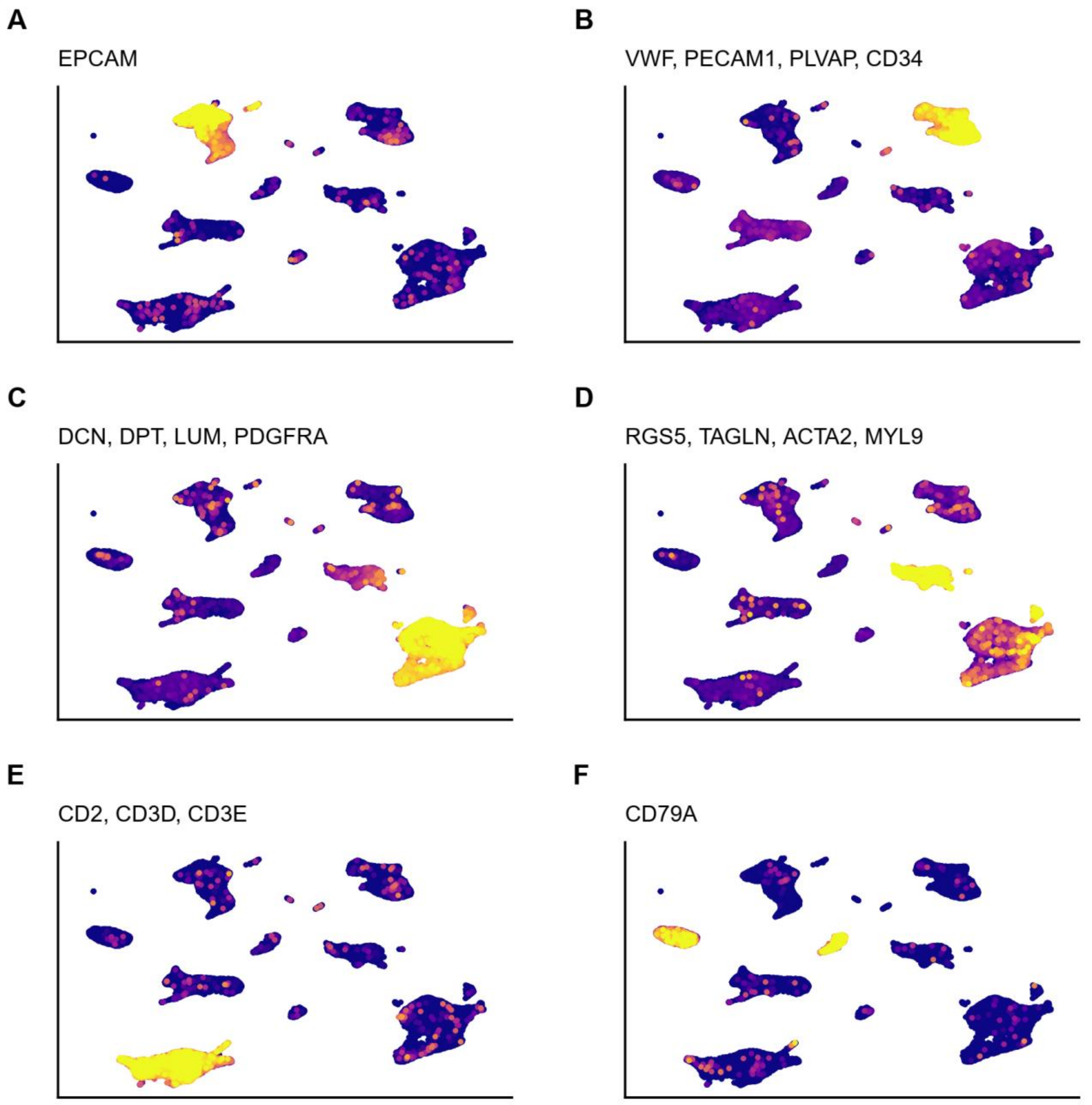

Supplementary Figure 4. Expression of selected markers are shown on a UMAP representation of the whole data set, related to Figure 1. A-F Yellow color indicates high expression of the gene(s) indicated on top, *blue indicates low expression.*

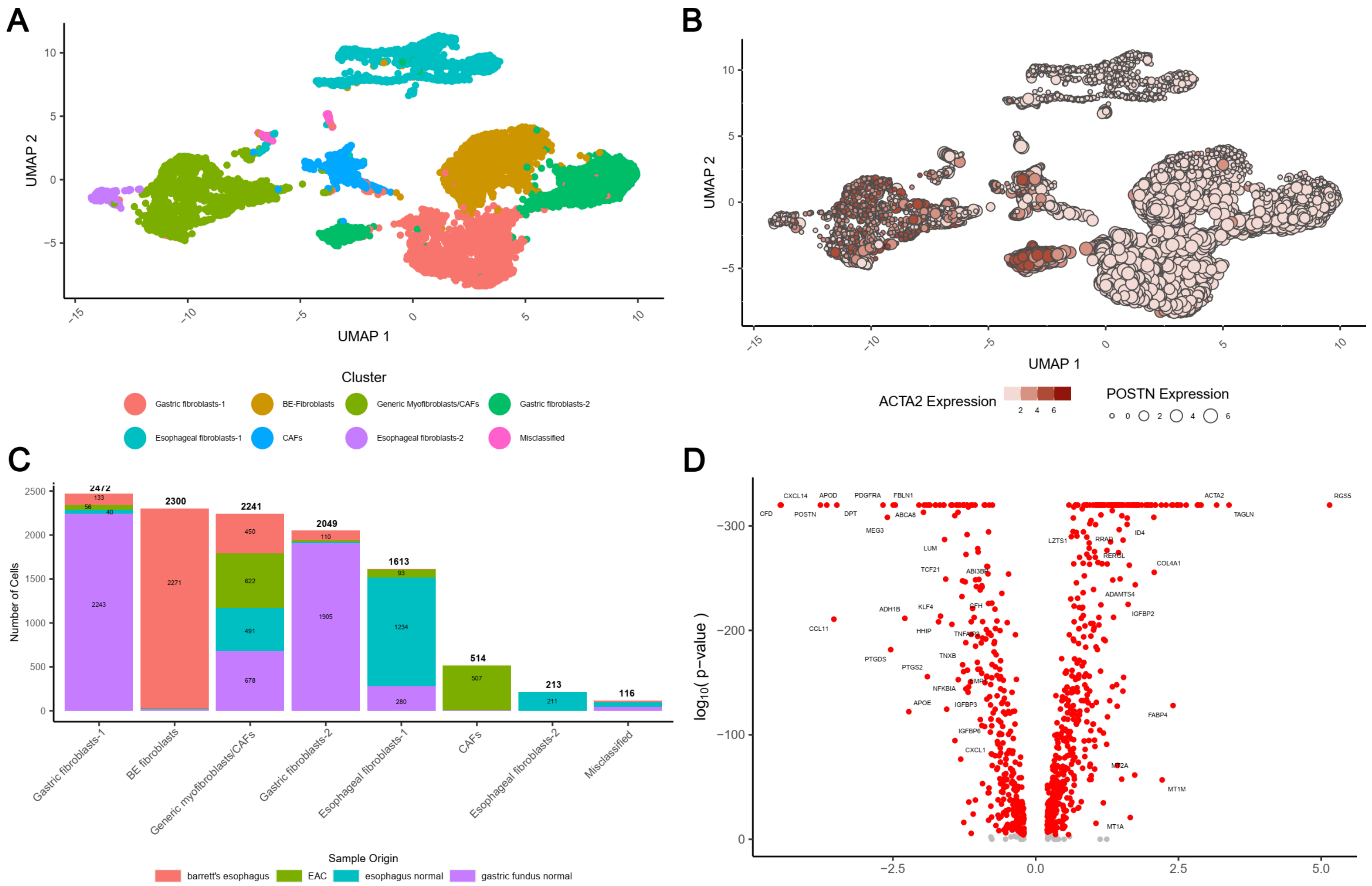

Supplementary Figure 5 Non-epithelial cell types are characterized and compared, related to Figure 1. A UMAP representation of fibroblasts, myofibroblasts and CAFs after cluster annotation. **B** ACTA2 vs. POSTN expression in the fibroblast-like cells. **C** Contribution of sample origins to fibroblast-like species. **D** Volcano plot of DEA of the ACTA2-high and the POSTN-high expressing clusters.

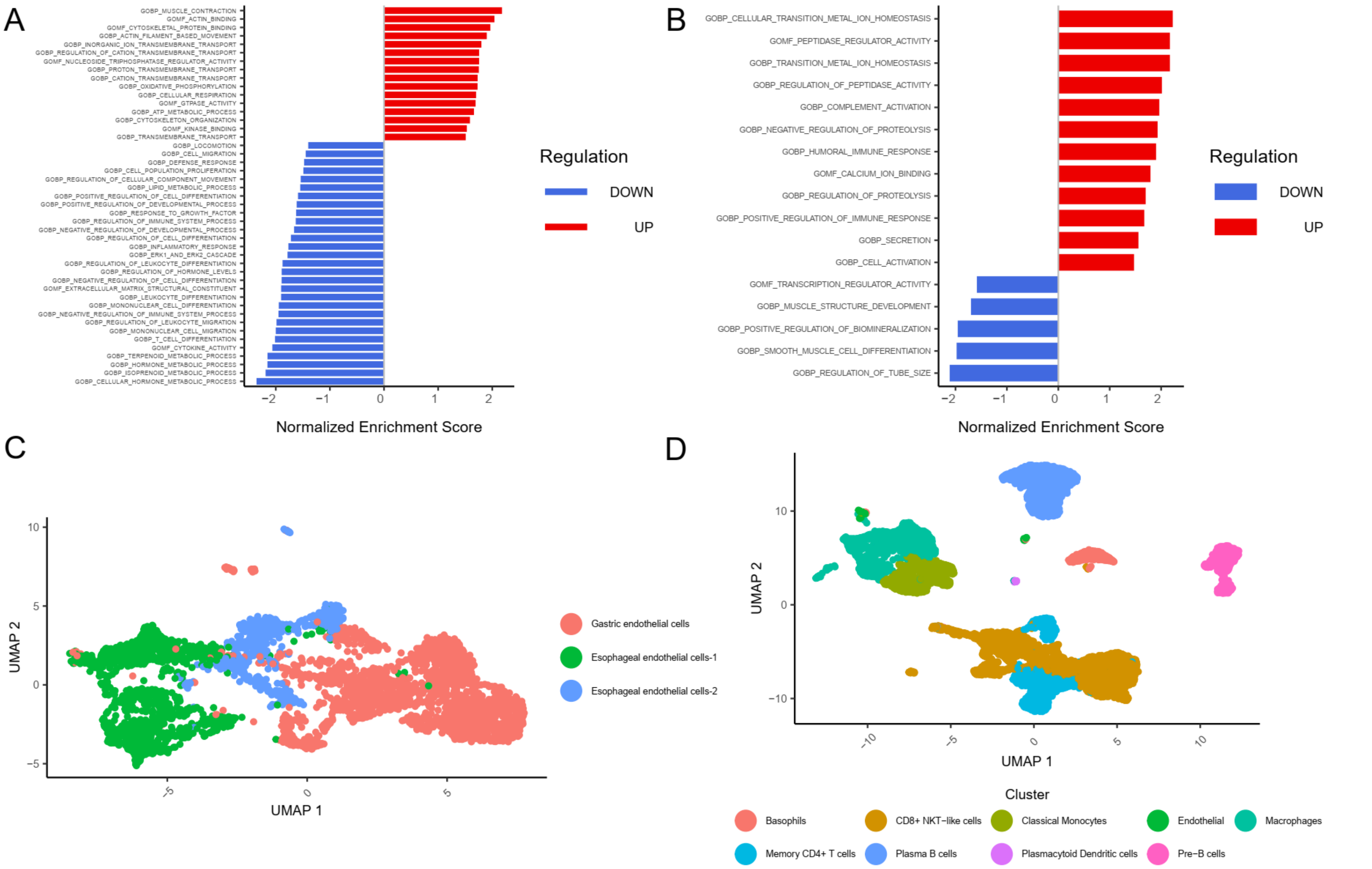

Supplementary Figure 6. Non-epithelial cell types are characterized, related to Figure 1. A GSEA of myofibroblasts vs. fibroblasts. **B** GSEA of esophageal fibroblasts-1 vs. other normal fibroblasts. **C** UMAP representation of the endothelial cells after cluster annotation. **D** UMAP representation of immune cells after cluster annotation.

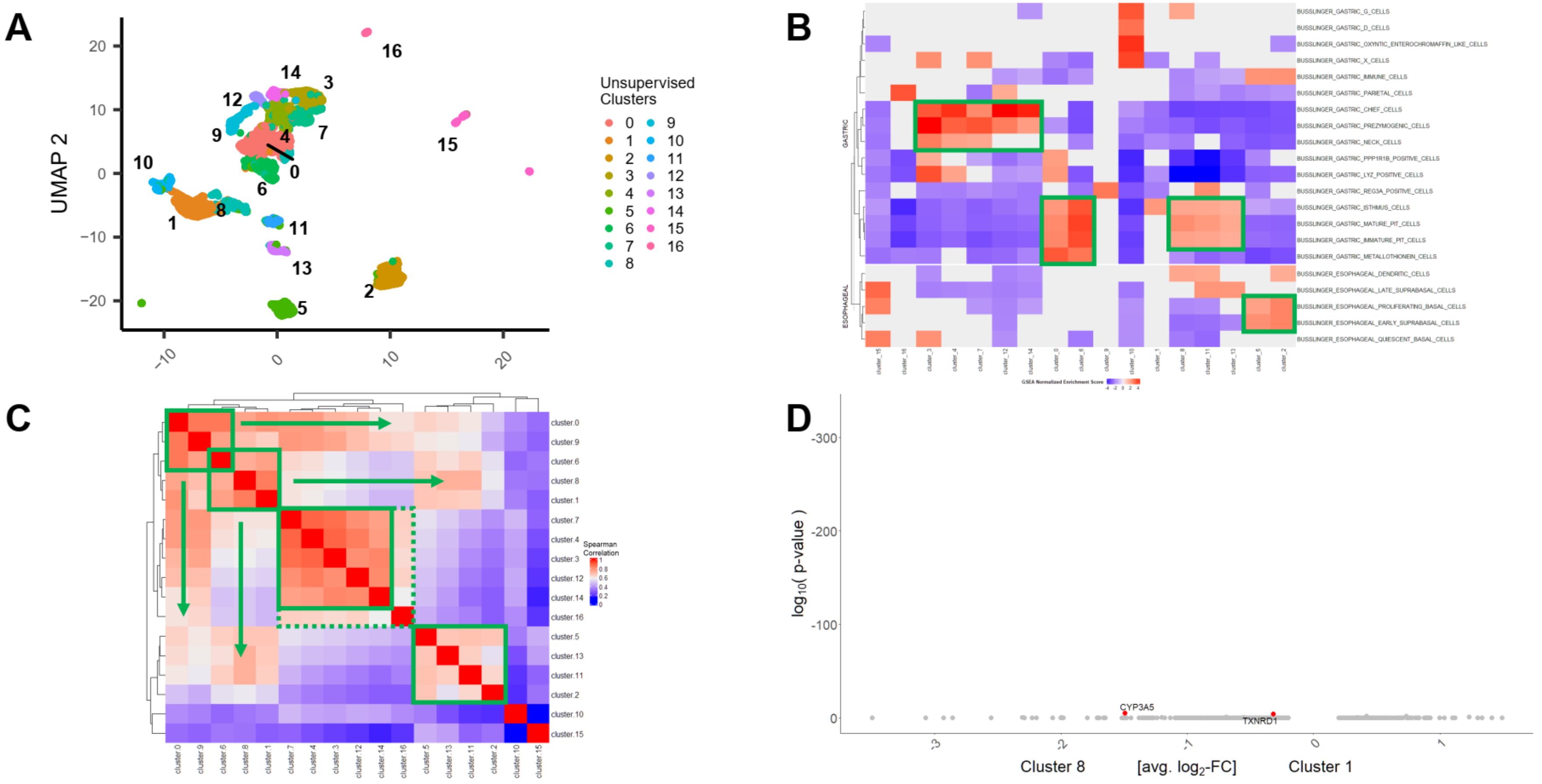

Supplementary Figure 7 Supplementary plots of the subset of epithelial cells indicate similarities, related to Figure 2. A UMAP representation of the epithelial cells with initial cluster color-coding. **B** GSEA of the initial clusters using the terms from Busslinger et al. [3]. **C** Spearman correlation matrix of initial clusters. **D** DEA of cluster 1 vs. 8 exemplifying minimal expression differences in correlated clusters.

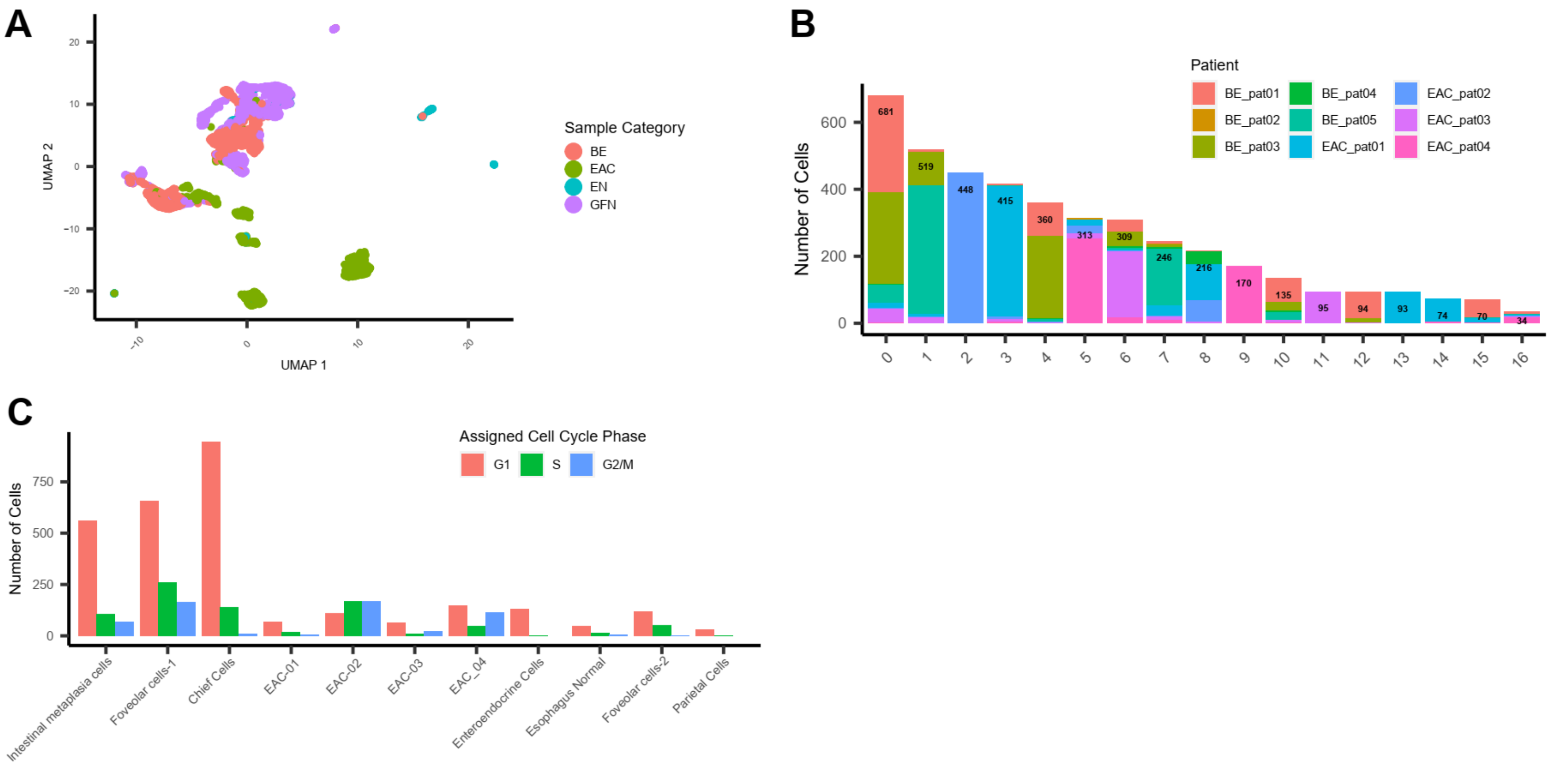

Supplementary Figure 8. Supplementary plots of the subset of epithelial cells indicate tissue origin and cell cycle phase, related to Figure 2. **A** UMAP representation of epithelial cells regarding sample origin. EN, esophagus normal; GFN, gastric fundus. **B** Composition of initial epithelial clusters regarding patient origin. **C** Absolute number of cells in each of the cell phases from the merged epithelial clusters.

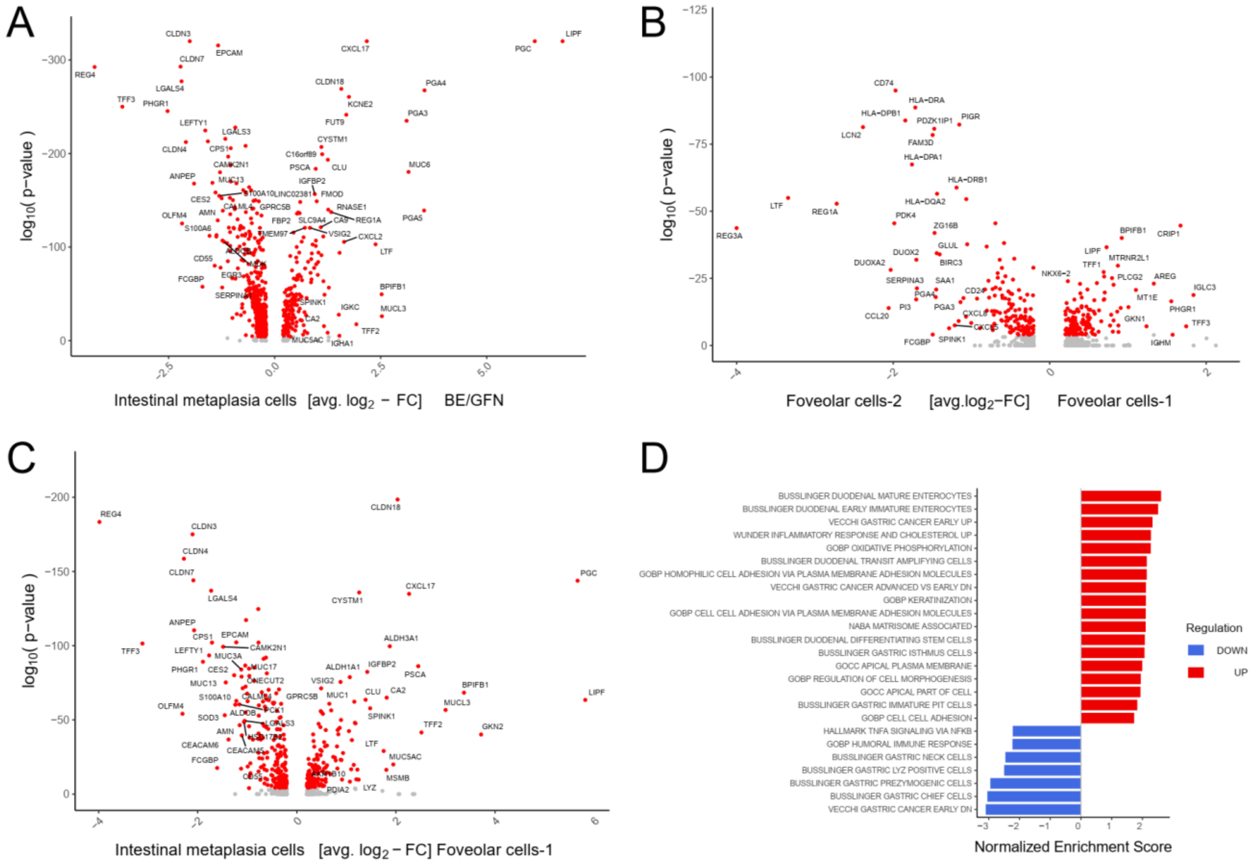

Supplementary Figure 9. Differential expression analysis characterizes intestinal metaplasia cells, foveolar cells, and cells of the BE/GFN group, related to Figure 4. A Volcano plot of the differential expression between intestinal cells with cells of the BE/GFN group. **B** Volcano plot of the differential expression between foveolar cells-1 and foveolar cells-2. **C** Volcano plot of the differential expression between intestinal metaplasia and foveolar cells 1. **D** GSEA of **A** with “up” referring to enrichment in BE/GFN group und “down” to intestinal cells.

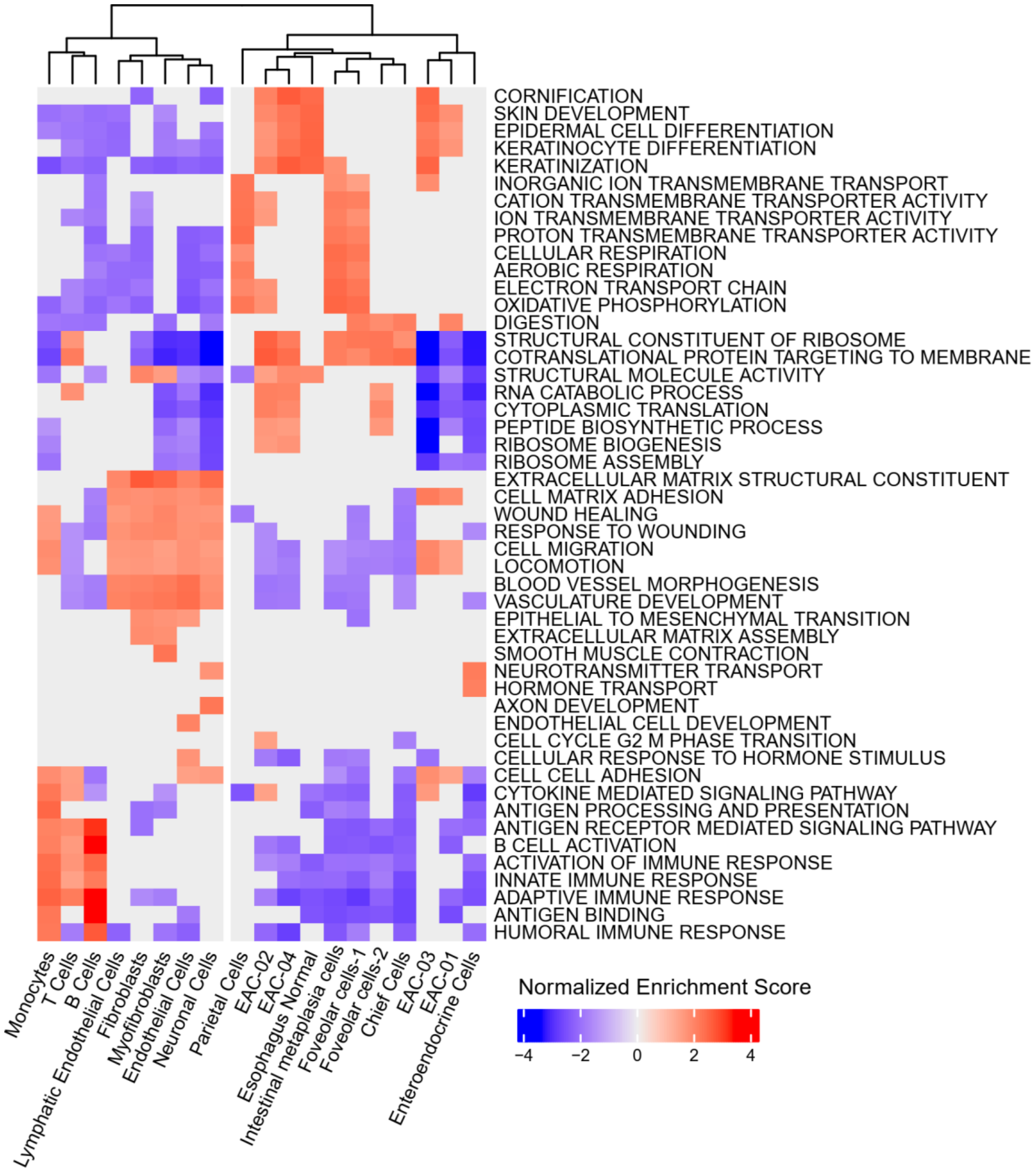

Supplementary Figure 10. GSEA of epithelial and malignant cells are shown in the context of non-epithelial cell types, related to Figure 4. Selected results of GSEA of the epithelial clusters and other cell types for balance. Only GO terms are displayed.

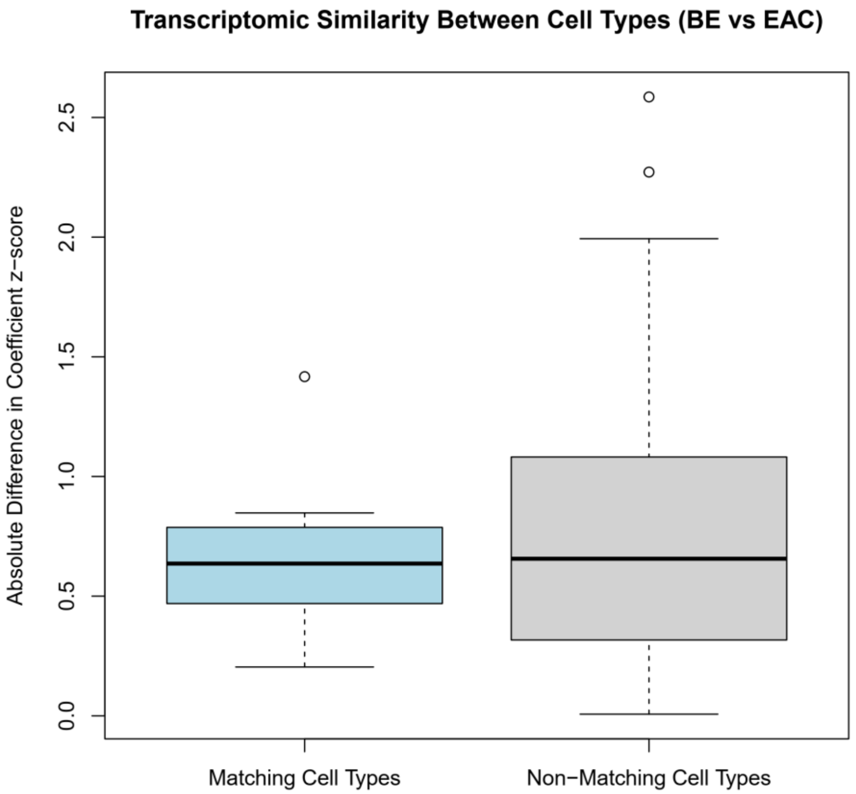

Supplementary Figure 11. LDSR coefficient z-scores of matching cell types from BE and EAC patients are correlating, related to Figure 5. Coefficient z-scores are significantly lower for matching cell populations, e.g. intestinal metaplasia cells from BE vs. EAC patients compared to non-matching cell populations, e.g., fibroblasts of BE patients vs. endothelial cells of EAC patients ($p = 0.02$ for the F-test and $p = 0.03$ for Levene's test), indicating that shared cell populations retain relatively stable GWAS enrichment profiles across BE and EAC conditions, despite the presence of cancer.

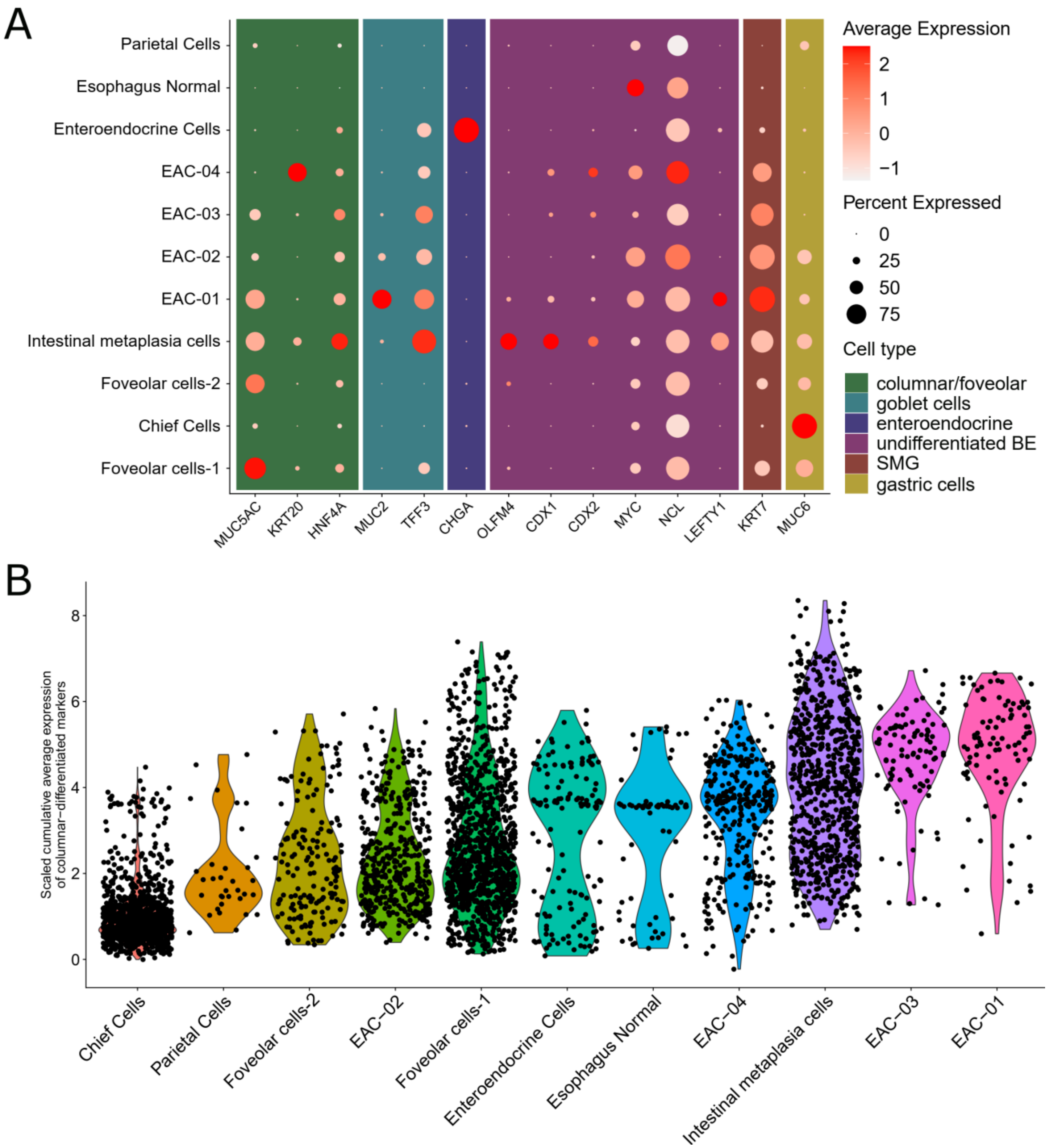

Supplementary Figure 12. Expression of marker genes of columnar-differentiated cells of Nowicki-Osuch et al. [4] are enriched in intestinal metaplasia cells, related to Figure 5. A Comparison and expression of marker genes from Nowicki-Osuch et al. in our epithelial cell types [4]. **B** Marker genes derived from Supp Table 7 of Nowicki-Osuch were tested for expression in cells of the present study with every point representing one cell. Expression data was scaled, 0 centered with standard deviation of 1 and values represent a cumulative average per gene and cell.

Supplementary References

1. Pasello, G., Agata, S., Bonaldi, L., Corradin, A., Montagna, M., Zamarchi, R., Parenti, A., Cagol, M., Zaninotto, G., Ruol, A., et al. (2009). DNA copy number alterations correlate with survival of esophageal adenocarcinoma patients. Mod Pathol *22*, 58-65. 10.1038/modpathol.2008.150.
2. Frankell, A.M., Jammula, S., Li, X., Contino, G., Killcoyne, S., Abbas, S., Perner, J., Bower, L., Devonshire, G., Ococks, E., et al. (2019). The landscape of selection in 551 esophageal adenocarcinomas defines genomic biomarkers for the clinic. Nat Genet *51*, 506-516. 10.1038/s41588-018-0331-5.
3. Busslinger, G.A., Weusten, B.L.A., Bogte, A., Begthel, H., Brosens, L.A.A., and Clevers, H. (2021). Human gastrointestinal epithelia of the esophagus, stomach, and duodenum resolved at single-cell resolution. Cell Rep *34*, 108819. 10.1016/j.celrep.2021.108819.
4. Nowicki-Osuch, K., Zhuang, L., Jammula, S., Bleaney, C.W., Mahbubani, K.T., Devonshire, G., Katz-Summercorn, A., Eling, N., Wilbrey-Clark, A., Madissoon, E., et al. (2021). Molecular phenotyping reveals the identity of Barrett's esophagus and its malignant transition. Science *373*, 760-767. 10.1126/science.abd1449.
